# An Analytical Model of Interlaminar Fracture of Polymer Composite Reinforced by Carbon Fibres Grafted with Carbon Nanotubes

**DOI:** 10.3390/polym10060683

**Published:** 2018-06-19

**Authors:** Feng Xu, Hong-Yuan Liu, Xusheng Du

**Affiliations:** 1 School of Astronautics, Northwestern Polytechnical University, Xi’an 710072, China; xufeng@nwpu.edu.cn; 2 Center for Advanced Materials Technology (CAMT), School of Aeronautics, Mechanical & Mechatronic Engineering J07, The University of Sydney, Sydney, NSW 2006, Australia; hong-yuan.liu@sydney.edu.au; 3 Institute of Advanced Wear & Corrosion Resistance and Functional Materials, Jinan University, Guangzhou 510632, China

**Keywords:** polymer composites, laminates, interlaminar fracture, analytical modelling, CNTs@CFs

## Abstract

An analytical model was developed to study the interlaminar fracture behaviour of polymer composite reinforced by carbon fibres grafted with carbon nanotubes. Delamination properties, such as load with displacement or crack (*R*-curve) and toughness with crack (*G_R_*-curve), can be obtained from this model. The bridging laws presented, based on the CNT pullout mechanism (CNT pullout from polymer matrix) and the CNT sword-in-sheath mechanism (CNT breakage), were incorporated into the proposed analytical model to investigate the influence of the structure of CNT growth onto CFs (CNT@CFs) on delamination properties. The numerical results showed that different toughening mechanisms led to different features of *G_R_*-curves, *R*-curves, and load with displacement curves. Parametric study demonstrated that strengthening the CNT@CF interface resulted in significant improvement in toughness. Further, it was found that elastic deformation of CNTs played an important role in the toughness improvement in the CNT sword-in-sheath mechanism, but no such role was evident in the CNT pullout mechanism.

## 1. Introduction

Carbon fibre-reinforced polymer (CFRP) composite laminates have been widely used in weight-critical structures, such as aircraft, spacecraft, and racing cars, due to their excellent mass-specific mechanical properties [1,2]. However, poor interlaminar toughness has become an important limiting factor in practical structural applications. To overcome the deficiencies in through-thickness strength, different solutions have been developed, including z-pinning, stitching, and weaving technology [3]. Unfortunately, significant reduction in in-plane properties (stiffness and strength) has been reported due to damage from the insertion of reinforcements in the through-thickness direction [4]. One effective method for improving delamination resistance without degrading in-plane properties is the incorporation of carbon nanotubes (CNTs) into the polymer matrix region [5]. Actually, the in-plane mechanical properties of composites could be even enhanced through the alignment of CNT in matrix [6]. However, as a result of the poor dispersion, lack of alignment, and damage to CNTs during processing [7], no significant improvement in interlaminar toughness of the resulting CFRP laminates has been achieved [8]. The challenge remains, therefore, to develop a new solution to facilitate practical applications of such composites as reliable and robust structural material.

In recent years, there has been successful perpendicular growth of CNTs onto fibre (CNT@CF) using various chemical catalysation techniques such as chemical vapour deposition (CVD) [9] and a flame synthesis method [10]. Remarkable improvement in toughness in mode I double cantilever beam (DCB) delamination with CNTs grown on fibres has been obtained by several research groups [11,12,13]. Various mechanisms, such as the pullout mechanism [14,15] and the sword-in-sheath mechanism [16,17], are considered responsible for the increase in toughness. These toughening mechanisms may work separately or simultaneously, contributing more or less to fracture toughness. As a complement to the experimental studies, some theoretical studies have recently been developed to provide a more effective way of investigating the influence of the CNTs@CF on the mode I delamination toughness of laminated CFRP composites. Tong et al. [18] presented an analytical model based on a stress-intensity factor solution to study the parametric effect on toughness improvement in CNT@CF hybrid composite laminate. Their results indicated that the density and length of CNTs, as well as interfacial frictional shear stress, were important parameters affecting delamination toughness. Blanco et al. [19] presented a simpler closed-form expression based on J-integral theory to investigate the toughening effect of different CNT bridging laws. Their investigation indicated that the improvement in toughness resulting from the pullout mechanism was greater than that from the sword-in-sheath mechanism. They found that consistent increases in CNT-matrix interfacial stress and CNT length could switch the CNT-matrix pullout mechanism to a CNT sword-in-sheath mechanism, finally leading to less toughness improvement. Yang et al. [20] developed a modified model based on the toughness model of Blanco et al. using established quadruple bridging laws of CNT pullout by the molecular dynamic (MD) simulation method. Their numerical results revealed that the CNT diameter and length and the loading conditions greatly affected CNT pullout behaviour from the polymer matrix, consequently leading to different toughness values. However, the aforementioned models did not characterize the influence of CNT toughening mechanisms on the entire behaviour of full *G_R_* curves (initial and steady state) and other detailed delamination properties, which are useful references in many applications. Moreover, CNT elastic deformation in the bridging laws was neglected by both Blanco et al. [19] and Tong et al. [18], with the possible result of underestimating the predicted toughness, especially in the CNT sword-in-sheath mechanism. 

More recently, in the case of CNTs grown on fibres, a new mechanism for the detachment of CNTs from fibres (CNT detachment mechanism) was presented and the grafting strength between the grown CNT and the fibre was successfully measured by Du et al. [10,21], followed by other groups [22,23]. Romanov et al. [24] found that the CNTs@CFs hybrid structure could reduce the stress concentration at the CF-matrix interface and transfer more stress at the crack face to the matrix resin zone within the CFRP laminate. Further, Du et al. [21] found that strong adhesion between CNTs and CF could lead to higher interfacial shear strength [25] and interlaminar fracture toughness in hierarchical epoxy composites. However, none of the aforementioned models considered the influence of CNTs@CFs and the mechanical properties of the CNT (failure strength and modulus) on the delamination properties of the CFRP laminates when the CNTs were pulled out from the matrix or broken in situ. 

In the present study, an analytical model is established to provide more details of the DCB delamination process. More importantly, realistic architectures (CNTs@CFs) and CNT elastic deformations are considered in the modelling process. Delamination crack length vs. load (*R*-curves), delamination crack opening displacement vs. load, and interlaminar fracture toughness of DCB samples with the influence of CNT toughening mechanisms on them are reported and compared to experimental results.

## 2. Critical Toughness of Mode I Delamination of Laminate Reinforced by CNT@CF

**Figure 1:**
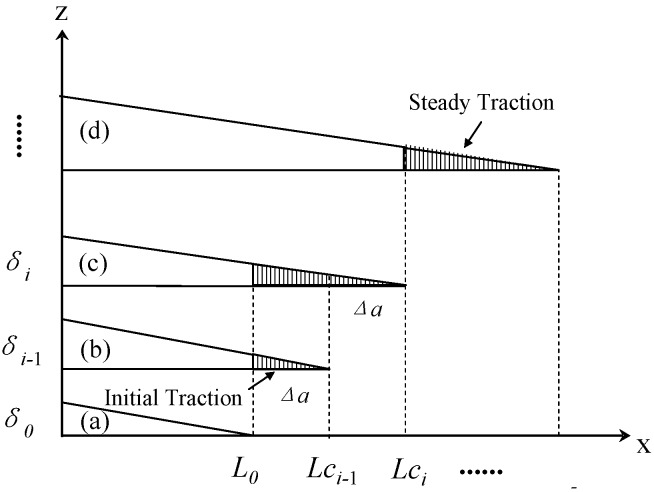
Diagram for CNT bridging and traction stages.

Figure 1 shows two CNT traction stages when a delamination crack propagates. The first stage is the “initial traction stage”, in which the length of the CNTs’ traction zone increases from zero with the crack propagation until the first single CNT adjacent to the initial crack length *L*_0_ is completely pulled out from the matrix (see Figure 1a–c). The second stage is the “steady traction stage”, in which the CNTs’ traction zone remains unchanged while the crack propagates (see Figure 1c,d).

**Figure 2:**
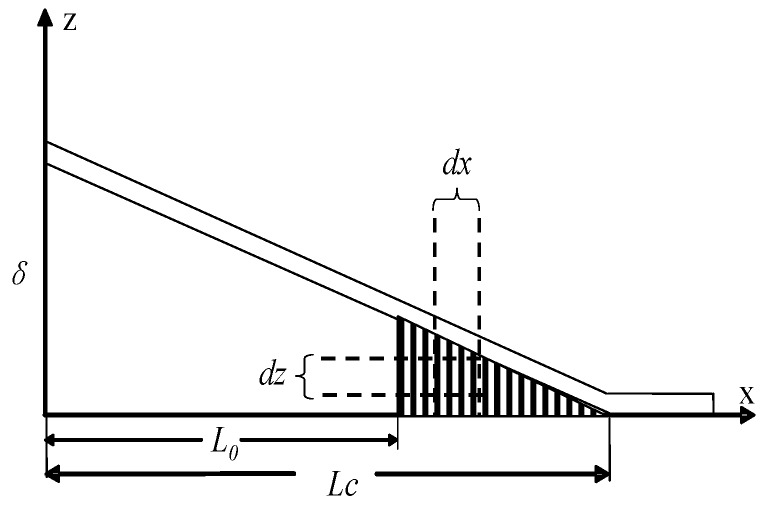
Typical CNT traction zone of DCB specimen.

Figure 1b,c represents typical adjacent intermediate steps in the initial traction stage. A typical CNT traction zone can be found in Figure 2, from which the dissipated energy *dU_CNT_* at the infinitesimal element *dx* region can be established as:

(1) $$\begin{array}{cc} dU_{CNT}=N_{CNT}\int_{0}^{u(x,Lc)}P(z)dz & L_{0}<x< \end{array}Lc$$

where *P*(*z*) is the bridging law for a single CNT, that depicts the relationship of the force and displacement when CNTs resist cracking; *u*(*x*,*Lc*) is the opening displacement of the DCB, that is also the CNT traction length when the variable *x* is located in the CNT traction zone (*L*_0_ < *x* < *Lc*); *N_CNT_* is the number of CNTs at the infinitesimal element *dx* region and can be obtained as follows:

(2) $$N_{CNT}=\frac{4Vwdx}{\pi D^{2}}$$

where *V* and *D* are the CNTs@CF grafting density and CNT diameter respectively, *w* is the width of the DCB specimen. At the i^th^ crack delamination intermediate step (see Figure 1c), the total dissipated energy *U_i_* originates from the sum of the dissipated energy of each CNT in the traction zone (from *L*_0_ to *Lc_i_*) and can be expressed by introducing Equation (2) into Equation (1):

(3) $$U_{i}=\{\begin{array}{cc} 0 & i=1\\ \frac{4Vw}{\pi D^{2}}\int_{L_{0}}^{Lc_{i}}\int_{0}^{u(x,Lc_{i})}P(z)dzdx & i>1 \end{array}$$

According to the definition of the energy release rate of a laminate beam [26], the critical toughness *G_i_* at the i^th^ crack delamination intermediate step is expressed from the upper and lower part of the DCB specimen:

(4) $$G_{i}=\{\begin{array}{cc} G_{0} & i=1\\ G_{0}+2\frac{U_{i}-U_{i-1}}{w\Delta a} & i>1 \end{array}$$

where *G*_0_ is the fracture toughness of the unmodified DCB specimen. Introducing Equation (3) into Equation (4) and considering the relationship of *Lc_i_* = *L*_0_ + *i*Δ*a*, where Δ*a* is the length of an intermediate step of crack propagation as shown in Figure 1, critical toughness, *G_i_* (*i* > 1), can be expressed as:

(5) $$\begin{array}{l} G_{i}=G_{0}+2\frac{4Vw}{\pi D^{2}}\cdot \frac{1}{w\Delta a}(\int_{Lc_{i}-i\Delta a}^{Lc_{i}-(i-1)\Delta a}\int_{0}^{u(x,Lc_{i})}P(z)dzdx\\ +\int_{Lc_{i}-(i-1)\Delta a}^{Lc_{i}}\int_{0}^{u(x,Lc_{i})}P(z)dzdx-\int_{Lc_{i-1}-(i-1)\Delta a}^{Lc_{i-1}}\int_{0}^{u(x,Lc_{i-1})}P(z)dzdx) \end{array}$$

when Δ*a* tends to be infinitesimal, Equation (5) can be simplified as:

(6) $$\begin{array}{lll} G_{i} & = & G_{0}+2\frac{4Vw}{\pi D^{2}}\cdot \underset{\Delta a\to 0}{\lim}\frac{1}{w\Delta a}\int_{Lc_{i}-i\Delta a}^{Lc_{i}-(i-1)\Delta a}\int_{0}^{u(x,Lc_{i})}P(z)dzdx\\ & = & G_{0}+2\frac{4Vw}{\pi D^{2}}\cdot \underset{\Delta a\to 0}{\lim}\frac{1}{w\Delta a}\int_{L_{0}}^{L_{0}+\Delta a}\int_{0}^{u(x,Lc_{i})}P(z)dzdx\\ & = & G_{0}+2\frac{4V}{\pi D^{2}}\cdot \underset{\Delta a\to 0}{\lim}\frac{d(\int_{L_{0}}^{L_{0}+\Delta a}\int_{0}^{u(x,Lc_{i})}P(z)dzdx)}{d\Delta a}\\ & = & G_{0}+\frac{8V}{\pi D^{2}}\int_{0}^{u(L_{0},Lc_{i})}P(z)dz \end{array}$$

Equation (6) represents the general expression for predicting the initial critical toughness of mode I delamination reinforced by CNTs@CFs if the CNTs@CF grafting density *V*, diameter *D*, initial crack length *L*_0_, crack propagation length *Lc_i_*, CNT pullout length *u*(*L*_0_,*Lc_i_*), and CNT bridging laws *P*(*z*) can all be determined. When the crack opening displacement *u*(*L*_0_,*Lc_i_*) increases with the crack length *Lc_i_* and finally tends to be the maximum CNT pullout length $l_{CNT}$ (including the CNT-matrix pullout length *l_p_* or the CNT length of the walls sliding out *l_f_*; more detail of *l_p_* and *l_f_* will be presented in Section 3), the saturation traction state is achieved (see Figure 1c,d) and the steady-state toughness can be expressed as:

(7) $$G_{R}=G_{0}+\frac{8V}{\pi D^{2}}\int_{0}^{l_{CNT}}P(z)dz$$

The closed-form Equations (6) and (7) give us predicted initial and steady toughness with crack propagation (*G_R_* curves), respectively, as a function of the bridging law *P(z)* and CNT pullout length *l_CNT_*. Thus, a closed-form expression for the prediction of toughness due to different toughening mechanisms can be detailed if the CNT toughening bridging laws are determined. 

## 3. CNT Toughening Mechanisms and Their Bridging Laws in DCB Delamination

The bridging law for a single CNT constitutes the foundation for determining the toughness model of CNTs@CFs reinforced CFRP composites. It is generally accepted that the toughness enhancement due to CNTs in the interlaminar region is attributed mainly to the CNT pullout process [18,19,20], involving (i) CNT pullout from polymer matrix; and (ii) CNT sword-in-sheath pullout. Thus, the various complex bridging laws, based on the Lawrence shear-lag theory [27] or nonlinear shear cohesive theory [28], have been continuously developed with the aim of good agreement with CNT pullout experimental curves [29]. The former bridging laws were established by the FE model [30], molecular dynamic model [20], and nonlinear theoretical model [29], and potentially increase computational cost or complicate the modelling process. However, in our concern to reduce the numerical computation to the minimum and facilitate application of the final proposed toughness model, linear analytical bridging laws with direct relationships and clear form can be established after a few assumptions are made:

(1) The CNTs are treated as unidirectional and straight multi-wall nanotubes without defects, and they are radially and uniformly grown on the surface of carbon fibres and then embedded into epoxy matrix to produce the CNTs@CFs hybrid structure.
(2) Based on assumption (1), the effects of any curvature [31], direction misalignment [32], stochastic failure [33], and bending [34] of individual CNTs on CNT pullout behaviour are minor. Therefore, these effects are ignored in this theoretical study.
(3) The CNT-matrix interfacial shear strength (normally 10–47 MPa [15,18,19]) is less than the matrix yield strength (>60 MPa [35,36]), thus, no matrix failure occurs and the possible matrix damage before CNT pullout is ignored in this numerical study.

### 3.1. CNT Matrix Pullout Laws

Pullout of individual CNTs from polymer matrix in direct experiments [14,15] and molecular dynamic simulations [20,37] confirms the three stages of CNT deformation and its corresponding force-displacement curves [31,38]. 

**Figure 3:**
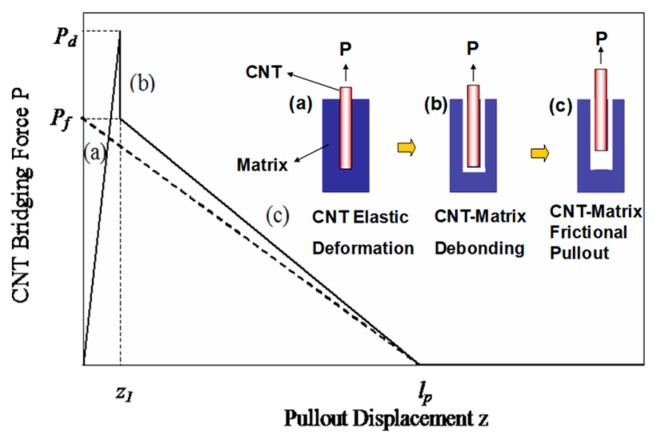
The typical bridging laws and schematic diagram for pullout mechanism.

As shown in Figure 3 by a solid line: (a) the CNT bonding stage when the interface between the CNT and its surrounding matrix is fully bonded. Due to the axial elastic stretching of CNT, the bridging force is linearly increased to the peak load *P_d_*; (b) the CNT debonding stage when the interfacial shear stress between the CNT and matrix exceeds the interfacial shear strength, the bridging force drops steeply with the increasing of displacement due to the full interface fails simultaneously; and (c) the CNT pullout stage when the interfacial debonding completes, the CNT is progressively pulled out from the matrix, and the frictional force *P_f_* gradually decreases to zero due to the interfacial frictional stress caused by radial compression stress at the interface between the fibre and matrix [18]. Thus, the corresponding bilinear bridging law for the above CNT deformation can be expressed as:

(8) $$P=\{\begin{array}{cc} \frac{P_{d}}{z_{1}}z & 0\leq z\leq z_{1}\\ P_{f}-\frac{P_{f}}{l_{p}-z_{1}}(z-z_{1}) & z_{1}\leq z\leq l_{p} \end{array}$$

In Equation (8), *l_p_* is the actual pullout length of the CNT from the matrix, which is shorter than the entire CNT length *L_CNT_*. *P_d_* is the debonding force for the full debonding process and, thus, can be written as: $D\pi \tau_{d}L_{CNT}$, in which $\tau_{d}$ is the CNT/matrix interfacial bonding strength. *P_f_* is the maximum frictional force applied on the CNT pullout length *l_p_*, *P_f_* can be determined as: $D\pi \tau_{f}l_{p}$, in which $\tau_{f}$ is the CNT/matrix interfacial frictional stress. Since the applied stress $\sigma_{a}$ along the full length of the CNT during elastic deformation is expressed as $4L_{CNT}\tau_{d}/D$, which is used as the stress-based criterion, the CNT fractures instead of pulling out of the matrix if the applied stress $\sigma_{a}$ is greater than the CNT strength $\sigma_{CNT}$. Using the stress-based criterion, the CNT maximum stretching displacement *z*_1_ can be further expressed as: $4L_{CNT}^{2}\tau_{d}/E_{CNT}D$, in which *E_CNT_* is the CNT Young’s modulus. To facilitate use of the CNT pullout bridging law, as indicated in Figure 3 by the dotted line, a simpler linear bridging law is proposed if the CNT elastic deformation is ignored (*z*_1_ = 0):

(9) $$P=\begin{array}{cc} P_{f}-\frac{P_{f}}{l_{p}}z & 0<z<l_{p} \end{array}$$

The pullout length *l_p_* is the critical parameter that determines the calculated energy release rate based on the CNT-matrix interfacial frictional process. In Blacon’s modelling analysis [19], half the length of the CNT was chosen as the maximum pullout length in the pullout mechanism when determining fracture toughness, in which the effect of the CNTs@CFs’ hybrid structure was not considered for toughness prediction. 

**Figure 4:**
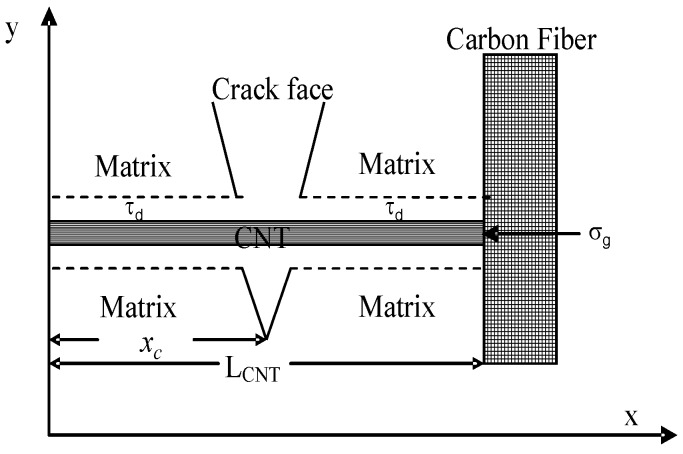
Diagram of CNT pullout process in CNT@CF structure.

In our model, the grafting tension stress in the CNTs@CF hybrid structure is considered. In the case of the CNT pullout from polymer matrix, the CNT can be pulled out from any crack position *x_c_* along the CNT length direction, as shown in Figure 4. However, based on the interfacial bonding strength *τ_d_* and the CNTs@CFs’ grafting tension stress *σ_g_*, a force balance expression can be established if a certain value of the crack position *x_c_* is reached, where 0 < *x_c_* < *L_CNT_*. In this case, letting the CNT pullout length *l_p_* = *x_c_*, we have:

(10) $$\pi Dl_{p}\tau_{d}=\pi D(L_{CNT}-l_{p})\tau_{d}+\sigma_{g}\pi {\left(D/2\right)}^{2}$$

Transforming Equation (10) gives the maximum pullout length *l_p_*:

(11) $$l_{p}=\{\begin{array}{cc} \frac{L_{CNT}}{2}+\frac{\sigma_{g}D}{8\tau_{d}} & 0\leq \sigma_{g}<\sigma_{c}\\ L_{CNT} & \sigma_{g}\geq \sigma_{c} \end{array}$$

In Equation (11), the pullout length *l_p_* can be theoretically obtained if the CNT/matrix interfacial bonding strength *τ_d_*, the CNTs@CFs grafting tension stress *σ_g_*, the CNT full length *L_CNT_*, and the CNT diameter *D* are given. It should be pointed out, however, that the CNTs@CFs grafting tension stress *σ_g_* theoretically cannot exceed the CNT@CFs grafting strength *σ_c_* [10] and, thus, the pullout length *l_p_* is normally less than the full length of the CNT. 

The establishment of Equation (11) implies that the existence of grafting tension stress *σ_g_* in CNTs@CFs hybrid structure potentially further increases the CNT pullout length in the pullout mechanism, and the CNT@CF grafting strength *σ_c_* at maximum is theoretically equal to the CNT failure strength *σ_CNT_*. It should be noted that the maximum pullout length *l_p_* is half of the full CNT length when the CNT is separated from CF (*σ_g_* = 0); this is consistent with the pullout length used in Blanco’s model, where the CNT@CF structure was not considered.

### 3.2. CNT Sword-In-Sheath Pullout Laws

Like the CNT pullout law from polymer matrix, the bridging law for the sword-in-sheath mechanism has been considered with both CNT elastic deformation and friction processes according to experimental observation and measurement [16,17]. 

**Figure 5:**
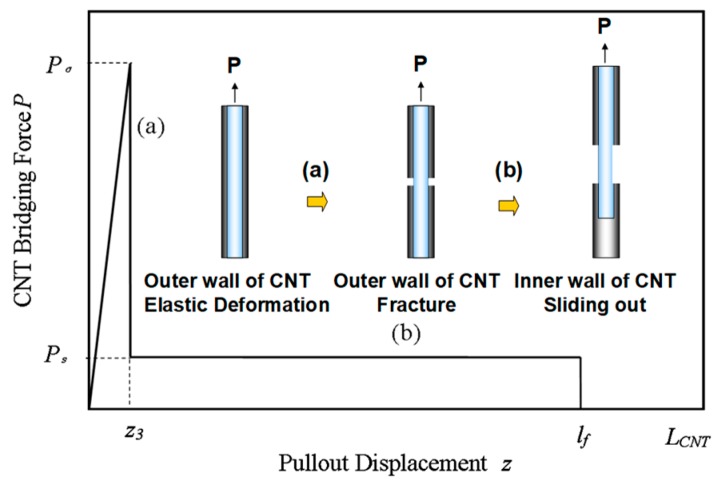
Typical bridging law and schematic diagram for sword-in-sheath mechanisms.

As shown in Figure 5, (a) with an increase in the applied load, the outermost wall of the CNT experiences elastic deformation until fracture when the applied stress *σ_a_* along the length of the CNT is greater than its strength *σ_CNT_*; then (b) the rest of the inner CNTs are pulled or frictionally slide out and the sliding force Ps is proved to be constant, based on observation of the experimental curves [16,39] and molecular dynamics analysis [40]. Previous work [39] has shown that the sliding force is independent of the overlapping length between the inner and outer walls of the CNT during the pull-out process and only depends on the diameter and layer numbers of multi-wall CNTs. The sliding force estimated here originates mainly from the van der Waals (vdW) attraction between the multiwall CNT interlayer. 

On the basis of the above analysis, we assume a bilinear bridging law that includes both the linear CNT elastic deformation (stage (a)) and the CNT sliding process (stage (b)):

(12) $$P=\{\begin{array}{cc} \frac{P_{\sigma }}{z_{3}}z & 0\leq z\leq z_{3}\\ P_{s} & z_{3}\leq z\leq l_{f} \end{array}$$

where *l_f_* is the pullout length of the CNT inner wall, *P_σ_* is the CNT fracture force and can be expressed as $\sigma_{CNT}\pi {\left(D_{CNT}/2\right)}^{2}$ , and *z*_3_ is the corresponding displacement and can be given as $L_{CNT}\left(\sigma_{CNT}/E_{CNT}\right)$. It should be pointed out that the sliding force is generally caused by the vdW force and the frictional force between the walls, where the vdW force is dominant in nearly defect-free CNTs with high crystallinity, whereas the frictional force may be significant when referring to defects or chemical crossing-linking [41] of the CNT. To simplify the analysis, the full expression of the sliding force *P_s_* is not detailed here due to its complexity, and the maximum CNT pullout length lf is assumed to be the full CNT length *L_CNT_*, due to the weak sliding force between the CNT walls. 

## 4. Toughness Models and Numerical Study

### 4.1. CNT Pullout Model

The steady-state improved toughness consisting of CNT elastic deformation $∆G_{ps}^{e}$ and CNT pullout from polymer matrix $∆G_{ps}^{f}$ can be given by introducing Equations (8) and (11) into Equation (7):

(13) $$\Delta G_{ps}=\Delta G_{ps}^{e}+\Delta G_{ps}^{f}=\frac{16V\tau_{d}^{2}L_{CNT}^{3}}{ED^{2}}+\frac{4V\tau_{f}}{D}{\left(\frac{L_{CNT}}{2}+\frac{\sigma_{g}D}{8\tau_{d}}\right)}^{2}$$

Further, a simpler toughness model from the linear law can be obtained by introducing Equations (9) and (11) into Equation (7):

(14) $$\Delta G_{ps}=\Delta G_{ps}^{f}=\frac{4V\tau_{f}}{D}{\left(\frac{L_{CNT}}{2}+\frac{\sigma_{g}D}{8\tau_{f}}\right)}^{2}$$

Similarly, the initial-state enhanced toughness can also be obtained if the pullout length *l_p_* is replaced by $\left(L_{0},Lc_{i}\right)$. It is clear that the toughness model described by Equation (14) can be converted to Blanco’s model [19] if the CNT@CF grafting tension stress *σ_g_* = 0. It should be pointed out that the proposed CNT pullout models include CNTs@CFs grafting tension stress as a key parameter, which may influence the improvement in toughness of the DCB specimen (to be detailed in Section 5.2). However, the grafting tension stress of CNTs@CFs has not been considered in established models [18,19,20]. 

### 4.2. CNT Sword-In-Sheath Model

The steady-state improved toughness arising from the CNT elastic deformation $\Delta G_{ss}^{e}$ and the sliding out process between the CNT walls $\Delta G_{ss}^{f}$ can be given by introducing Equation (12) into Equation (7):

(15) $$\Delta G_{ss}=\Delta G_{ss}^{e}+\Delta G_{ss}^{f}=\frac{8V}{\pi D^{2}}(\frac{\sigma_{CNT}^{2}L_{CNT}\pi D^{2}}{8E_{CNT}}+L_{CNT}(1-\frac{\sigma_{CNT}}{E_{CNT}})P_{s})$$

Normally, *σ_CN_* ≪ *E_CNT_* (the mean CNT failure strength and modulus are 30 GPa [17] and 1000 GPa, respectively [41]). Therefore, a simpler model based on the CNT sword-in-sheath model can be approximately expressed as:

(16) $$\Delta G_{ss}=\Delta G_{ss}^{e}+\Delta G_{ss}^{f}=\frac{VL_{CNT}\sigma_{CNT}^{2}}{E_{CNT}}+\frac{8VL_{CNT}P_{s}}{\pi D^{2}}$$

Unlike the CNT pullout models, the proposed CNT sword-in-sheath model considers the CNT elastic deformation behaviour and, thus, includes key parameters, such as CNT failure strength *σ_CNT_* and modulus *E_CNT_*, which also potentially influence the toughness improvement of the DCB specimen (to be detailed in Section 5.1). However, CNT elastic deformation behaviour has not been considered in established models or in [18,19,20], in which the investigators claim that the energy contribution during CNT elastic deformation is minor and, thus, can be neglected. 

### 4.3. Validation of Toughness Models 

The models proposed above were validated by a comparison with recent experimental results presented by Du et al. [21] and the predicted results of Blanco’s pullout model. In Du’s experimental work, carbon nanotubes were deposited in situ onto the plain woven CF fabric (Inter-Turbine Advanced Logistics Pty Ltd, Banyo, Australia) according to our recent developed flame synthesis method [21]. The plain woven carbon fabric applied with the nickel chloride catalyst precursor (NiCl_2_, 0.2 mol/L) was mounted on a metal frame and inserted into the core of the flame at 500–700 °C. In the process, such an atmosphere not only provides the carbon species for the growth of CNTs and prevent the CFs from combustion, but also reacts with the nickel chloride directly, leading to the formation of Ni catalyst particles for CNT growth on CFs. The good grafting between CNTs and CFs was observed and could be attributed to the diffusion of metal catalyst particles into the CF surface and/or carbon bonding between CNTs and CFs [25]. Plain woven fabrics with or without CNTs were utilized as the main reinforcement in the CFRPs. Araldite-F (diglycidyl ether of bisphenol A, Huntsman) and piperidine (Sigma-Aldrich, St. Louis, MO, USA) in a weight ratio of 100:5 were used as the matrix. Laminates having 16 plies of woven fabrics were prepared by the hand lay-up method. A 25 μm thick polyimide film was inserted in the mid-plane of the laminates to act as the initial crack. The laminates were then wrapped with bleeders and release film within a vacuum bag, first vacuumed in a chamber for 0.5 h, followed by curing in a hot-press at 120 °C for 16 h under a pressure of 200 kPa. The fibre volume fraction in the final composite laminates was 58%. The DCB specimen was 150 mm long, 20 mm wide, and 3 mm thick, and the initial crack length was 48 mm. The composite is assumed to have flexural modulus $E_{b}=20\;\mathrm{GP}$. The flexural rigidity of the beam was calculated by $EI=E_{beam}^{x}wh^{3}/12$. The initial crack length in the laminate is 48 mm and the delamination propagation is 20 mm. The critical toughness for the unmodified laminate is *G*_0_ = 500 J/m^2^, obtained from our previous work [21], and it is assumed that this value remains unchanged during the delamination process. The baseline parametric values for our CNT pullout model and Blanco’s pullout model, unless otherwise noted, are: *L_CNT_* = 2 μm, *V* = 3.14%, *D* = 50 nm, and *σ_f_* = *σ_d_* = 47 MPa [15]. The CNT@CF grafting tension stress *σ_g_* = 1 GPa [10] is the additional input for our CNT pullout model. It should be pointed out that the CNTs’ grafting density *V* given above was determined by the distance (250 nm) between two adjacent CNTs in a square array based on the observation of the photographs in [21]. Furthermore, the baseline values for our CNT sword-in-sheath model, unless otherwise noted, are *L_CNT_* = *L_f_* = 2 μm, *V* = 3.14%, and *D* = 20 nm, values based on the grown CNTs produced previously in our group [10,21]. Other parameters, *σ_CNT_* = 30 GPa [17], *E_CNT_* = 1000 GPa [42], and *P_S_* = 100 nN [16], that were not given in [21], are taken as mean values in [16,17,42] for the input of both proposed toughness models. 

**Figure 6:**
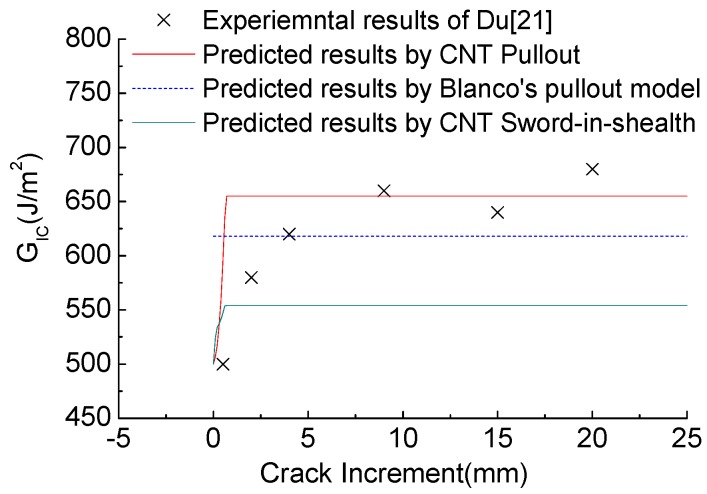
Comparison between predicted and experimental results for toughness models.

Figure 6 provides comparisons between predicted results (the presented CNT pullout model, the CNT sword-in-sheath model, and Blacon’s pullout model [19]) and experiments. As Figure 6 shows, the experimental *R*-curve has two steps. The first step is characterized by the increase in *G_IC_* from the critical toughness value (500 J/m^2^) to the toughness value (650 J/m^2^) at rather small crack increments. The first step is well predicted by our presented CNT pullout model, which seems to be more comprehensive than Blacon’s pullout model that could only predict a plateau in toughness. The predicted toughness value of our CNT pullout model, that is higher than that of Blacon’s pullout model, derives from the additional consideration of the CNTs@CF grafting tension stress and the CNT elastic deformation in our bridging laws. Meanwhile, a second step occurs in which the toughness value of *G_IC_* plateaus at around 650 J/m^2^ (20 mm crack increment length). However, our proposed model can only give the unique plateau of the toughness value arising from the constant CNT traction zone with delamination. Moreover, the steady-state *G_R_* value (solid green line) predicted by the sword-in-sheath toughness model is much lower than the values obtained from the pullout models, confirming that the CNT pullout from the matrix rather than the CNT sword-in-sheath model was the dominant toughening mechanism in [21]. 

### 4.4. Characterization of Delamination Properties 

**Figure 7:**
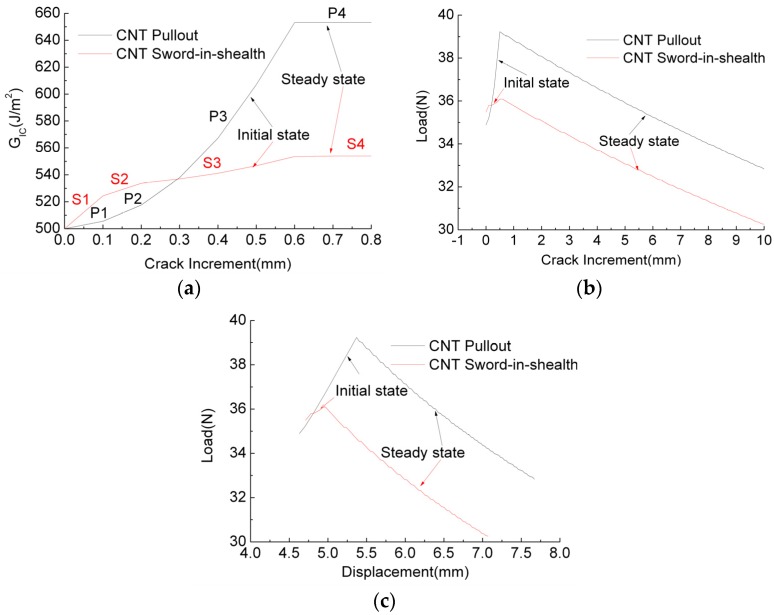
Delamination properties prediction: (**a**) typical *G_R_* curves; (**b**) typical R curves; and (**c**) typical load with displacement.

To better understand how the micro-CNT bridging laws (CNT pullout and CNT sword-in-sheath) affect macro-delamination properties, based on the same parameters as those used in Section 4.3, as shown in Figure 7, the typical *G_R_* value vs. crack length curve was calculated and analysed by the proposed toughness model and the corresponding CNT bridging laws, after which the typical R curves and load vs. displacement curves were recalculated though the calculated toughness values based on classic beam theory [43]. For simplicity of analysis, the initial load-displacement curves for elastic deformation of the beam are not given in Figure 7c.

Figure 7a shows two typical full *G_R_* curves (toughness with crack) obtained from CNT pullout and CNT sword-in-sheath bridging laws, respectively. For *G_R_* curves in the initial state, as indicated by arrows, it can be seen that the CNT pullout bridging laws cause the slope of the curve (black line) to increase, so that a concave curve is produced, whereas the CNT sword-in-sheath bridging laws lead to a curve with a decreasing slope (red line), producing a convex curve. Thus, the different laws lead to *G_R_* curves with different features, providing a useful reference for distinguishing CNT failure mechanisms when a macro DCB test is conducted. These two full *G_R_* curves can each be divided into four stages, representing the different CNT traction states with crack propagation: (S1/P1) elastic deformation for all CNTs; (S2/P2) CNTs elastic deformation and partial pullout/sliding out; (S3/P3) CNT partial pullout/sliding out and complete pullout/sliding out; and (S4/P4) CNT complete pullout/sliding out. It is found that CNT pullout-based *G_R_* values become much higher than CNT sword-in-sheath-based *G_R_* values when the CNT pullout/sliding out occurs from the S2/P2 stage to the S4/P4 stage. These results indicate that CNT pullout from the matrix dissipates much more energy than the CNT sliding out process. 

Figure 7b,c gives the typical *R* curves (load with crack) and load with displacement curves, respectively, each of which contains both the initial state and a steady state, as indicated by the arrows. It is found that all the crack resistance loads increase during the initial state and then decrease at a steady rate with crack length/displacement. From comparisons of Figure 7a with Figure 7b,c, it is concluded that higher toughness values originate from the high resistance load (see Figure 7b,c) and high displacement (see Figure 7c). Particularly in Figure 7c, where the curves represent the raw data obtained from the DCB experiment, it should be noted that the crack begins to propagate before the maximum load is reached, and the maximum load does not start to decrease until all the CNTs in the traction zone have completely pulled out/slide out during the steady crack propagation stage. 

## 5. Parametric Study and Discussion

Many publications [18,19,37] have reviewed the effect of certain parameters on toughness improvement. These parameters have included CNT volume fraction, CNT length and diameter, CNT/matrix interfacial strength, and outer/inner-walls sliding frictional stress. Most of those parameters are also included in our proposed toughness models. The effects of these parameters on toughness are not discussed again here as similar trends are obtained. To satisfy real situations, CNT elastic deformation has been included in the CNT bridging laws and CNT@CF grafting tension stress has been considered in CNT@CF architectures, both of which have been incorporated into toughness models, regarded as the modification of Blanco’s model. In the following subsections, therefore, the elastic mechanical properties of CNTs and the grafting tension stress of CNT@CFs are studied respectively and baseline parameters for the simulation are still the same as those in Section 4.3, unless otherwise stated. 

### 5.1. Effect of CNT Elastic Deformation on Toughness

**Figure 8:**
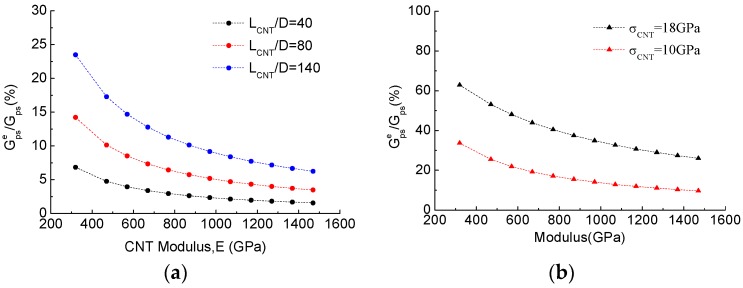
Effect of CNT elastic deformation on energy contribution in different CNT toughening mechanisms: (**a**) CNT pullout; and (**b**) CNT sword-in-sheath.

To investigate the significance of CNT elastic deformation on the contribution of steady toughness, as shown in Figure 8, the ratios $∆G_{ps}^{e}/∆G_{ps}$ and $∆G_{ss}^{e}/∆G_{ss}$ as the CNT elastic contribution were predicted as the functions of CNT elastic mechanical properties (CNT modulus *E_CNT_* and strength *σ_CNT_*), in which $G_{ps}^{e}$ and $G_{ss}^{e}$ are the CNT elongation energy from CNT pullout and CNT sword-in-sheath, respectively. 

Figure 8a demonstrates the effect of CNT modulus (from 320 GPa to 1470 GPa [40]) and CNT aspect ratio (*D* = 20 to 50 nm, *L_CNT_* = 2 μm) on the contribution of CNT elastic toughness under the CNT pullout mechanism. It is found that a decrease in the CNT modulus and an increase in the CNT aspect ratio lead to an increase in the elastic toughness of the CNTs (up to 25% of the total toughness), but that contribution is not significant. For example, CNT elastic toughness is responsible for less than 10% of the total toughness if the realistic values of 1000 GPa in the CNT modulus and 50 nm in the CNT diameter are used. That being the case, the energy contribution from CNT elastic deformation can be reasonably ignored and the toughness model described by Equation (14) can be used to predict CNT pullout toughness.

Figure 8b depicts the effect of CNT modulus (*E_CNT_* from 320 to 1470 GPa) and CNT failure strength on the contribution of CNT elastic toughness under the CNT sword-in-sheath mechanism. The CNT failure strength was assumed to be 10 and 18 Gpa, recalculated from the stress-based criteria based on the parameters *D_CNT_* = 20 nm, *σ_d_* = 47 MPa, *L_CNT_* = 2 μm. It is evident that a decrease in the CNT modulus and an increase in CNT failure strength cause a significant increase in toughness under the CNT sword-in-sheath mechanism (more than 50% of the total toughness), indicating that CNT elastic deformation cannot be neglected in the assumption of CNT sword-in-sheath bridging laws. This argument differs from that in previous CNT modelling work [18,19], in which the authors claimed that the energy contribution of CNT elastic deformation was minor and could, thus, be neglected in their model. 

### 5.2. Effect of CNT@CF Grafting Stress on Toughness

**Figure 9:**
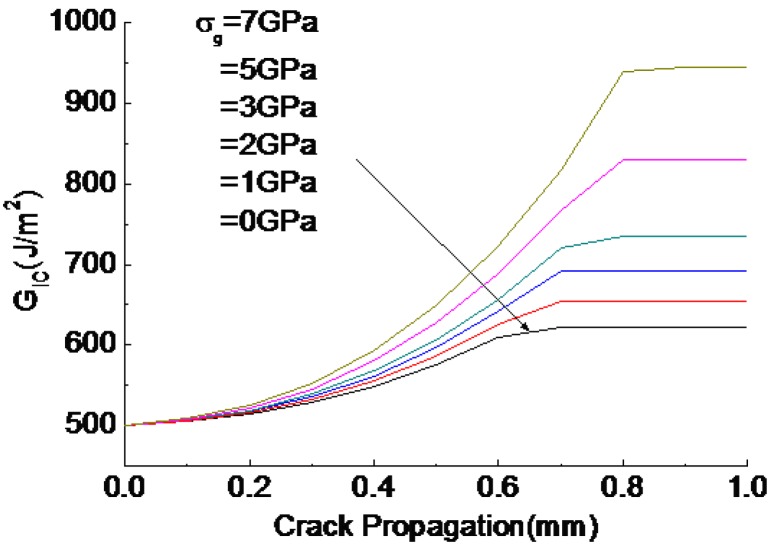
Effect of CNT@CF grafting tension stress on toughness improvement.

Figure 9 depicts toughness versus crack propagation for different values of CNT@CF grafting stress *σ_g_*, which is assumed to be 0 to 7 GPa as estimated by Equation (11). Clearly, an increase in *σ_g_* theoretically yields a significant increase in toughness (about 89% increase) compared to that of the unmodified laminate, a finding implies that strengthening the CNT@CF interface is an alternative efficient strategy for toughening laminate. However, the CNT@CF hybrid structure was not considered in the previous CNT pullout model [18,19]. 

## 6. Conclusions

An analytical model for DCB delamination of CFRP composites reinforced by CNTs@CFs was developed. Based on the available experimental observations, the CNT pullout and CNT sword-in-sheath bridging laws were assumed and incorporated into a proposed theoretical model to identify the influence of CNTs@CFs on the delamination behaviour of the CFRP laminates. The analytical model and parameter study provide the following conclusions:

(1) The present model can predict fully detailed delamination properties, such as crack resistance load versus displacement, crack resistance load versus crack propagation, and fracture toughness versus crack propagation in DCB testing.
(2) Different toughening mechanisms yield different delamination properties. Specifically, the CNT pullout mechanism results in a higher crack resistance load in R curves/load-displacement curves and leads to the concave curve type as the feature of *G_R_*-curves. In contrast, the CNT sword-in-sheath mechanism results in a lower crack resistance load in R curves/load-displacement curves and yields the convex curve type as the feature of *G_R_*-curves.
(3) Strengthening the CNT@CF interface is an efficient strategy to obtain significant toughness improvement. Furthermore, CNT modulus and failure strength have important roles in toughness improvement in the CNT sword-in-sheath mechanism, but such effects were not evident in the CNT pullout mechanism.
(4) Compared with other existing models, our model as presented in simulation provides better prediction and more information about DCB delamination behaviours.
